# Smart and Modern Thermoplastic Polymer Materials

**DOI:** 10.3390/polym10111211

**Published:** 2018-10-31

**Authors:** Andrea Pucci

**Affiliations:** Department of Chemistry and Industrial Chemistry of the University of Pisa, Via Giuseppe Moruzzi 13, 56124 Pisa, Italy; andrea.pucci@unipi.it; Tel.: +39-050-221-9270

*Smart* and *modern* thermoplastic polymer materials are defined as novel thermoplastic materials that are capable of responding to external stimuli through a macroscopic output in which the energy of the stimulus is transduced appropriately as a function of external interference. The bulk of these materials generally consists of polymers with plastic behavior and properties, while displaying a smart response through, for example, optical or electrical signals. A thermoplastic polymer is a plastic that becomes ductile or moldable above a threshold temperature and returns to a solid state upon cooling [1]. Most thermoplastics have a high molecular weight with macromolecular chains that are associated through intermolecular forces, which permits thermoplastics to be remolded into a variety of shapes and also enables them to lend themselves to recycling. The term *smart* implies that the material displays a response to an external stimulus and that this occurs in a controlled manner [2]. Research into *smart* thermoplastic polymers has increased greatly over the last few decades, principally owing to growing interest in upgraded materials for new and *modern* applications (Figure 1).

Inputs are generally triggered by scientific and research aspects that are often intertwined with societal issues and demands. Incentives for these demands are generally based on societal aspects such as economics (i.e., cost reduction) and sustainability. Thus, *smart* and *modern* thermoplastics are not limited only to scientific aspects but also intersect with societal relevance. The use of thermoplastic polymers with smart features is widespread, ranging from polymers for photovoltaics [3,4], to optical sensing [5,6], nanocomposites with graphitic fillers [7,8,9], thermoreversible materials [10,11], and many others. 

This Special Issue consists of nine original articles, one of these as a featured paper and authored by Prof. Ben Zhong Tang and collaborators. All the contributions highlight the latest research in the topic area and report on and discuss the preparation of *smart* thermoplastic polymers including polymer (nano)composites, their detailed characterization, and their ultimate applications in order to provide the most illustrative information. Many examples are devoted to the preparation of *modern* composites in which the graphitic filler consisting of pristine or chemically functionalized multilayer graphene or multi-walled carbon nanotubes is able to confer to the thermoplastic polymer advanced features such as improved thermal conductivity [12], electrical responsivity [13,14], mechanical reinforcement [15], and microwave attenuation [16]. In all these examples, a fundamental issue addressed by authors was the research of the highest interface interactions between the polymer matrix and the graphitic materials. The maximized interface between the components provided the highest output response in terms of thermal, electrical and mechanical characteristics that are intrinsic properties of the carbon-based fillers.

This Special Issue also reports the description of novel shape-memory epoxy-polyurethane co-networks by means of Diels–Alder couplings, i.e., one of the most intriguing and attractive areas of research on self-healing and recyclable polymer structures [17]. In this case, the (reversibly) crosslinked epoxy resin was rendered more flexible and ductile thanks to the interpenetrating network composed of polyurethane. An innovative and cost-effective network for wound-dressing applications was also proposed and based on mixtures of poly(vinyl alcohol)-poly(acrylic acid) [18]. The new material showed improved swelling, adhesion, and biocompatibility properties, while the mechanical properties of the poly(vinyl alcohol) remained mostly undamaged, making it suitable for a variety of biomedical applications.

Finally, polymeric materials with novel structures and unique properties and functionalities are described in this Special Issue. For example in the feature paper, functional poly(dihalopentadiene)s were stereoselectively synthesized, endowed by aggregation-enhanced emission features, and proposed for the sensitive detection of explosives [19]. Notably, the polymers are weakly emissive in dilute solutions but become highly emissive upon aggregation, demonstrating a unique phenomenon of aggregation-enhanced emission. Their nanoaggregates in aqueous media can serve as sensitive fluorescent chemosensors for the detection of explosives with a superamplification effect and a low detection limit. Furthermore, the synthesis of poly(phenylene methylene)s (PPMs) with high molar masses was reported by means of polymerization of benzyl chloride catalyzed with tungsten(II) compounds and subsequent fractionation [20]. Notably, four different tungsten(II) catalysts were successfully exploited for the polymerization, for which a strict temperature profile was developed. This study illustrated a new approach to synthesize PPM with hitherto unknown high molar masses, which opens the possibility of exploring *smart* applications, e.g., for temperature-resistant coatings, fluorescent coatings, barrier materials or optical materials.

By way of conclusion, I should like to thank all authors and reviewers for their invaluable contribution to this Special Issue. It is hoped that it can represent a ready reference for readers interested in *smart* and *modern* polymers and an effective stimulus to the academic and industry-related audience for the development of this topic in even more innovative classes of advanced materials. 

## Figures and Tables

**Figure 1:**
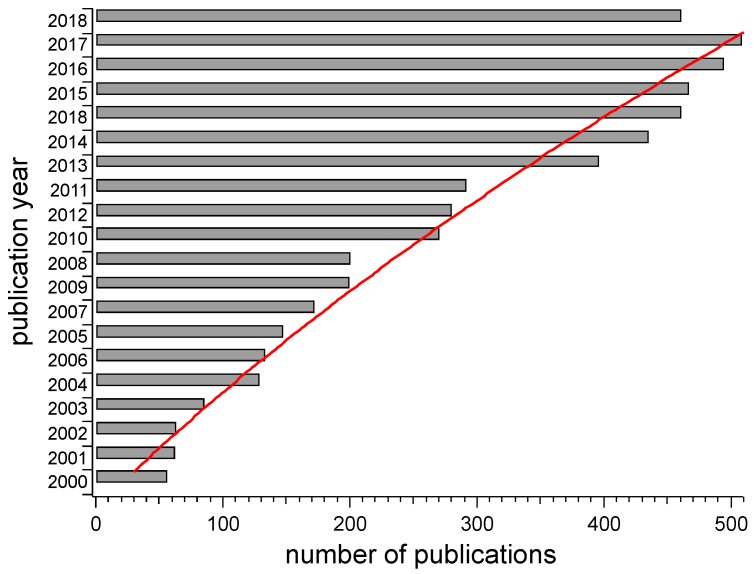
Number of publications in the field of *smart* and *modern* thermoplastics since 2000 and growing trend (red line). Source: CAS SciFinder^®^ Scholar, October 2018.
